# An Empirical Study on the Relationship between Causes of Teacher Examination Anxiety and Dimensions of Coping with Pre-Exam Anxiety: A Structural Equation Modelling Approach

**DOI:** 10.5334/pb.536

**Published:** 2020-08-13

**Authors:** Stella Shimave, Yagmur Cerkez, Engin Baysen

**Affiliations:** 1 Department of Guidance and Psychological Counselling, Near East University, North Cyprus, TR

**Keywords:** exam anxiety, avoidance, preparation, assessment, behaviours, beliefs

## Abstract

In recent years, examination anxiety among teachers assumes a critical sphere in the global academic environment. The causes of teacher examination anxiety in education have been reviewed by a few scholars. This shows that teacher examination anxiety and its impact on academic development are limited in research. Therefore, this study investigated the linear relationship between two self-report instruments – the causes of teacher examination anxiety and dimensions of coping with pre-exam anxiety. The study adopted a quantitative approach with three-hundred teachers from four secondary schools in Nigeria and twenty teachers from two secondary schools in North Cyprus participated in the survey. Also, a Structural Equation Modeling (SEM) was utilized for the analysis. The results of the study indicate that the two factors (teacher causes of exam anxiety and dimensions of coping with pre-exam anxiety) are interconnected. The results also indicate teachers’ preparation for examinations coupled with various dimensions of anxiety is a complex task that demands educational stakeholders to constantly improving on causes of examination anxiety and factors of pre-exam anxiety among teachers for better academic and ethical development.

Many comprehensive studies on teacher anxiety have received global attention in recent years in which has been linked with different educational and psychological behaviours within educational environments. It is frequently stated that anxiety is an experience that aroused sensitivity through worry and fearfulness, subjugated to test of individual’s knowledge and unique capability (Von der Embse, Jester, Roy & Post, 2018) and the desired outcome is to acknowledge the insistent call for a structured framework on anxiety within educational environments.

Accordingly, many leading educational psychologists (Green, Angoff & Encandela, 2016; Senler, 2016; Von der Embse, Schoemann, Kilgus, Wicoff & Bowler, 2017; Soni, & Kumari, 2017; Guven, 2017) have promptly investigated the possible causes and factors of pre-exam anxiety in educational domains. Although, many of the recent studies mostly focus on performance and evaluation of anxiety (e.g., Beilock, Gunderson, Ramirez, & Levine, 2010; Zeidner, 2010; Vaz, Pothiyil, George, Alex, Pothiyil, & Kamath, 2018; Novak & Tassell, 2017; Liou, Daly, Canrinus, Forbes, Moolenaar, Cornelissen & Hsiao, 2017; Garver, 2019; Timoštšuk, Kikas & Normak, 2016). while limited investigation on the relationship between causes of teacher test anxiety and other approaches of anxiety (Von der Embse et al., 2018; Maulana, Opdenakker & Bosker, 2016; Kurian, Ramanathan & Andrlic, 2018).

A comprehensive study by Dal, (2018) on the use of Big Five personality traits and narcissistic personality traits to predict pre-examination anxiety and self-confidence indicated that teachers’ high narcissism helps reduce anxiety and increases self-confidence before the examination. As is expected, the weight of an examination has led to anxiety about the situation and its consequences always undermine test performance (Ramirez & Beilock, 2011). Von der Embse et al., (2018) stated that for the past forty years, most research focus on test anxiety theory, related measurements, comparisons studies but limited studies on test anxiety variables across multiple studies.

Although, most empirical studies based their causes of teacher test anxiety on personal knowledge, ideological beliefs, and performance with just a few extensive studies on factors of pre-examination anxiety. For example, Boyacioglu and Kucuk (2011), suggested investigating unrealistic and irrational perspectives, fundamental beliefs, and philosophical thoughts caused by different factors of anxiety-like evaluation style, examination results, and psychological distress. The study stated that it is necessary to evaluate the influence of test-based accountability policies on teacher examination anxiety, instructional practices, and institutional policies for educator effectiveness (Von der Embse et al., 2017). The foregoing highlights the urgent need to investigate the causes and dimensions of coping with teacher anxiety and the purpose of this, is to investigate the causes of teachers’ test anxiety and dimensions of coping with pre-exam anxiety using teacher test anxiety and dimensions of coping with pre-exam anxiety scale to establish the relationship of anxiety and pre-exam anxiety among teachers.

In retrospect, the gap in the literature proposes an empirical approach on the relationship between the causes of teacher test anxiety and dimensions of coping with pre-exam anxiety among teachers using a structured linear relations and model fits to confirm teachers’ perspectives on examination anxiety, their strategic approach towards pre-exam anxiety.

## Teachers’ Examination Anxiety

Anxiety is a very sensitive and difficult issue that re-occur in all humans endeavour (Spielberger, 2010; Horwitz, Horwitz & Cope, 1986; Alpert & Haber, 1960; Reiss, Peterson, Gursky & McNally, 1986). Sucuoğlu, Nawaila and Shimave (2017) defined anxiety as an intense worry and self-consciousness that concern the immediate and anticipated circumstances. Horwitz (2010) termed anxiety as a multi-faceted and psychological science that consists of numerous types of anxiety-like traits, state, achievement, and facilitative-debilitative anxiety.

Also, Freud (1977), used the psychoanalytic theory of Sigmund Freud to explain that human nature and behaviour are controlled by factors such as irrational forces, unconscious motivations, biological and instinctual drives/forces. The theory proposes many constructs among is anxiety. This theory states that anxiety occurs as a result of the clash or conflict among other sets of constructs within the theory. Corey (2015) also stated that anxiety should comprise fear and grasp as a situation that triggers further effective action on anxiety. As a result, anxiety can be language anxiety, speech anxiety, social anxiety trait, and personality anxiety and it occurs in an accidental test-related contingency called examination anxiety.

Examination anxiety is defined as an anxiousness experience in an assessment or an examination period (Putwain, Woods & Symes, 2010). Lyness (2013) explained that examination anxiety is not the same as unsatisfactory performance in any specific test, and further based the issue on individual’s character. Individual’s anxiety can easily be controlled but difficult to manage other anxiety traits and is categorized into various forms based on the environment it occurs.

Zeidner and Mathews (2005) identified the component of examination anxiety as cognitive and affective-physiological factors. As cognitive factors occur when negative thoughts arise during the examination period – worries, irrelevant thinking and self-deprecating. While the affective-physiological factors occur based on the subjective perceptual experiences that involuntarily occur before the examination period such as lack of learning time and procrastination.

Further, many studies have established the fact that the anxiety during assessment cannot be defined as a threatening situation (Bodas & Ollendick, 2005; Hodapp, Rohrmann & Ringeisen, 2011) as it is usually influenced by personal characteristics before, during or after the evaluation process. For instance, Mealey and Host (1992) identified the habitual, irrelevant, negative thoughts during the assessment period as a primary cause of test anxiety. Hughes (2003) explained the causes and procedures of measuring language teachers’ skills and knowledge based on examination anxiety. Bekdemir (2010) stated that anxiety and embarrassment are positively affected by the teacher-focused approach of the test situation and academic stress arises from expectations and pressures (Ang & Huan, 2006; Putwain, Daly, Chamberlain, & Sadreddini, 2016). Saracaloglu, Dincer and Gerceker (2018) also analysed the relationship between teachers’ examination postponement attitude and anxiety using genders, score levels and time table as factors and the results implied that male experiences more academic postponement attitude and low examination anxiety scores than female level of anxiety.

As mentioned earlier, most studies on causes of teacher examination anxiety focus on personal characteristics of the test situation: gender factors, level of difficulty, worry, procrastination, stress, negative experiences, diverse forms of anxiety, competences and skill level (In’nami, 2006; Putwain et al., 2016; Oladipo & Ogungbamila, 2013). Therefore, examination anxiety affects learning, changes test performance, and causes academic self-esteem which practically repeals the aim of assessment and performance.

## Pre-Examination Anxiety

The three concepts of teachers’ examination anxiety that are widely used to are teachers’ pre-examination anxiety (Stöber, 2004), teachers’ anxiety during examination (Grills-Taquechel, Norton & Ollendick, 2010) and teachers’ post-examination anxiety (Davidson & Scutt, 1999). There are few intensive studies on key principles and models on teachers’ pre-examination anxiety within the academic environment (e.g., Bekdemir, 2010; Stöber, 2004; O’Carroll & Fisher, 2013).

The examination of teachers are mostly influenced by level of anxiety and their overall performance rather than teacher-oriented pre-exam anxiety (Núñez-Peña, Bono & Suárez-Pellicioni, 2015; Dal, 2018). Pre-examination anxiety occurs as a result of different factors before the examination. These include teacher’ personal traits before examination such as individual’s character, previous performance, incorrect choice of courses, location and time span of examinations, intimidation either short-term or long-term test anxiety, social pressure, worry, irrational thoughts, interference and lack of confidence (Putwain, Woods & Symes, 2010; Hoferichter, Raufelder, Ringeisen, Rohrmann & Bukowski, 2016). Moreover, lack of confidence and experiences (Machida, 2016).

According to Sucuoğlu, Nawaila and Shimave (2017), to reduce the effects of examination anxiety among teachers is to prime interest in their personal development, examination standard and evaluation system, excessive course load, lack of time to revise before the exam and lack of systematic studies (McKeachie & Svinicki, 2013). Moreover, Johnson, (1999) suggested that short-term and long-term anxiety by instructors can lead to regulate feelings (somatic) and thoughts (cognitive) that lead to worry/anxiety before the examination. Stöber, (2004) researched on the pre-exam anxiety conditions and uncertainty. The study measured individual dimensions of coping with pre-exam anxiety – task-orientation and preparation, seeking social support, irrelevant thinking, avoidance. The results of the study showed that different dimensions of test anxiety specify the relationships among diverse ways of coping with pre-exam anxiety.

However, the influence of task-orientation and preparation, seeking social support, avoidance and the level of test anxiety among students as well as teachers indicate that strategies are useful for pre-exam anxiety (Zeidner, 1996; Stöber, 2004; Putwain, Woods & Symes, 2010; Hyseni Duraku & Hoxha, 2018). Therefore, this study is not just concerned with the causes of teacher anxiety or dimensions of coping with pre-exam anxiety among teachers but also with the exploration of coping strategies for anxieties related to teachers’ evaluation system so as to improve their skills and competences.

## Research Context

The primary purpose for this study is the shared ground between the teachers’ anxiety and dimensions of coping with pre-exam anxiety in the educational domain. In preference, the Nigerian National Policy on Education (2014) mandates that all teachers should improve on their teaching qualification as part of the contribution towards educational development, teachers should be empowered for the process of educational growth, build-up acquisitions, and ethical qualities through improving the knowledge and skills needed to overcome anxieties at every level of educational development (Samuel & Adekunle, 2019; Chiu & Churchill, 2016). and that teachers must engage in in-service training to promote professionalism, anxiety and competencies in the educational system. Therefore, this study explores based on relationships between causes of teacher examination anxiety, and dimensions of coping with teacher pre-exam anxiety with respect to causes, approaches and perspectives (Hyseni Duraku & Hoxha, 2018, Putwain et al, 2016; Von der Embse et al., 2017; Contreras-Soto et al., 2019), with these, the following research questions are posed as follows:

1. What are the causes of teacher’s examination anxiety and dimensions of coping with pre-exam anxiety?
2. What are the relationships between causes of teacher examination anxiety and dimensions of coping with pre-exam anxiety?

## Method

### Respondents and procedure

To establish the objectives of the study, the researchers designed a questionnaire from previous studies that focuses on teachers’ anxiety towards examination and it was distributed to the target population of three-hundred teachers from four secondary schools in Nigeria and twenty teachers from two secondary schools in North Cyprus during the promotion examination exercise for in-service teachers. Data collected through a self-administered questionnaire survey method using a quasi-convenience snowball sampling method and A total of 320 respondents returned the questionnaire. We also obtained ethics approval from school authority and the ministry of education. The demographic respondent includes 93.7% (300) were Nigerian and 6.25% (20) were Cypriot teachers. Most of the respondents were female teachers 240 (75%) and Male (25%); (66.7%) of the teachers held an undergraduate degree, (29.5%) held a master’s degree and (3.8%) held a doctorate; just over 40.2% of the teachers had 10–20 years working experience and 21–35 years working experience.

### Measurement Instruments

This study developed measurement items for data collection including Twenty-one items with six constructs of the proposed model which were adopted from the previous studies. The measurement items were modified based on the study and the items were divided into two parts – teacher examination anxiety and dimensions of coping with pre-exam anxiety. The structured linear relationships and model fits were analyzed using JASP (Jeffrey’s Amazing Statistics Program) version 10.0.2.0.

#### Teacher exam anxiety inventory

The Teacher Test Anxiety (TTA) inventory was developed based on existing instruments (e.g., Fujii, 1993; Serrano-Pintado & Escolar-Llamazares, 2014; Mowbray, Jacobs & Boyle, 2015). The 12-item TTA consist of three items – general test worrying, test irrelevant thinking, and negative emotions as shown in Table 1. According to In’nami (2006), the instrument was obtained from the theory of test-relevant and test irrelevant thinking which was designed to measure the psychosomatic aspects of exam anxiety. The TTA was modified by In’nami (2006) with consistency reliability (CR) > 0.80.

**Table 1:** TTA and PEA items and factor loadings.

Items	Factor loadings

**Teacher Test Anxiety**	
***General test worrying (GTW)***	
Even when I am well prepared for a test, I feel very anxious about it.	0.900
I start feeling very uneasy just before examination	0.893
I wish examinations did not bother me so much	0.856
It seems to me that examination periods ought not to be made the tense situations which they are	0.896
***Test Irrelevant thinking (TIT)***	
Before the exams I find myself thinking of things unrelated to the actual course material	0.874
I find myself thinking of how much brighter the others are than I am	0.867
Before the exams I find myself thinking of the consequences of failing	0.850
Before the exams I sometimes wonder if I would ever get through it	0.861
***Emotion (EMO)***	
I get nervous before the exams	0.933
I feel uneasy before the exams	0.865
I feel my heart beating very fast before the exams	0.913
**PEA – Pre-Exam Anxiety**	
***Task-Orientation and Preparation (TOP)***	
I think about how I can best prepare for the exam.	0.819
I concentrate on how I am going to deal with the exam and, if necessary, let other things slide	0.850
I cut back on my leisure time to prepare for the exam	0.808
***Seeking Social Support (SSS)***	
I ask people who have had similar experiences what they did/would do in this situation.	0.837
I discuss my feelings with someone.	0.856
I try to get advice from someone about what to do.	0.846
***Avoidance (AVO)***	
I convince myself that it is not all bad.	0.967
I put thoughts of the exam out of my mind.	0.966
I try not to think about the exam.	0.951
I turn to other activities for diversion.	0.972

#### Pre-exam anxiety inventory

The 13-item Pre-Exam Anxiety Inventory (PEA) was developed by Stöber (2004) from the COPE inventory (Carver et al., 1989; Buchwald & Schwarzer, 2003). The study adopted three control items for the study measurement. The first item is task-orientation and preparation, emphasizing planning, and suppression of competing activities. The second item is seeking social Support with emphasis on instrumental reasons for assisting teachers in obtaining facts and the relationships among them. The third measure focuses on avoidance through anxiety suppression, denial, and trivialization as shown in Table 1. The PEA has been examined and validated with several dimensions of examination anxiety through different relationships and approaches to educational psychology The PEA was modified by Stöber (2004) with CR > 0.87.

## Results

To test the measurement model and the structural model, the study adopted a two-stage analytical procedure suggested by Hair, Ringle and Sarstedt, (2011). The purpose of this research is to assess the measurement model and measure the model fit to establish the correlations among the constructs based on the psychometric standard instruments for the analysis.

### Measurement Model

The purpose of the measurement model is to test the reliability, convergent validity and discriminant validity. The first step, exploratory factor analysis (EFA) to evaluate the reliability, convergent and discriminant validity of the proposed linear relationships. The convergent validity is the extent to which different measures of the same construct are significantly related to one another, while discriminant validity statistically measures factors that are distinct from each other (Anderson & Gerbing, 1988). Accordingly, Table 2 shows the Cronbach’s Alpha (α), Composite Reliability (CR), and Average Variance Extracted (AVE) – convergent validity of the measurement model with constructs used for measurement model (Fornell & Larcker, 1981). The results indicates that all the measurements are greater than the minimum levels. For CR, range between 0.773 and 0.938 and AVE between 0.682 and 0.929 which indicate an acceptable convergent validity.

**Table 2:** Convergent validity of the measurement model.

Construct	Composite Reliability (CR)	Average variance extracted (AVE)	Cronbach’s alpha(α)

GTW	0.938	0.786	0.910
EMO	0.892	0.817	0.888
TOP	0.773	0.682	0.767
AVO	0.976	0.929	0.975
SSS	0.786	0.546	0.714
TIT	0.890	0.745	0.886

Also, the discriminant validity statistically examined factors to determine the differences between each other (Anderson & Gerbing, 1988) and confirmed by checking the total square root of AVE if greater than the correlation of the constructs. As shown in Table 3, the discriminant validity of the measurement model result shows that the square root of the AVE is greater than the correlation of the constructs and with other constructs, which indicates a good discriminant validity.

**Table 3:** Discriminant validity of the measurement model.

Construct	GTW	EMO	TOP	AVO	SSS	TIT

GTW	**0.786**					
EMO	0.007	**0.817**				
TOP	0.023	0.161	**0.682**			
AVO	0.000	0.156	0.000	**0.929**		
SSS	0.027	0.053	0.272	0.000	**0.546**	
TIT	0.003	0.097	0.002	0.235	0.013	**0.745**

Squared correlations; AVE in the diagonal.

### Structural model and hypotheses testing

The second step after measurement model testing is to test linear relations and to determine the model fit of the linear relationships. Henseler, Ringle, and Sarstedt (2015) stated that model estimates of constructs must be greater than recommended values. Therefore, we tested linear relations and the model fit. The results in Table 4 indicates that the model provides a precise fit (Hair, et al., 2011). All the indicators of goodness of fit (X^2^/df = 2.44, RMSEA = 0.060, SRMR = 0.68, TLI = 0.967, CFI = 0.972) shows that the linear relationship between TTA and PEA is an acceptable model fit.

**Table 4:** Coefficient of the model.

Sample Coefficient	X^2^	df	p	X^2^/df	RMSEA	TLI	CFI

**Model fit**	439.877	180	<001	2.44	.060	.967	.972

Also, Table 5 summarizes the linear relationship between the constructs. The results show that general test worrying (β = 0.126, p < 0.001) is positive and significantly related to teacher task-orientation and preparation for the evaluation; emotion (β = 0.452, p < 0.001) is positive and significantly related to teacher task-orientation and preparation for the evaluation; and test irrelevant thinking (β = –0.195 p < 0.001) is negatively related to teacher’s task-orientation and preparation for the evaluation; emotion (β = 0.274, p < 0.001) is positively and significantly related to teacher avoidance for examination anxiety; and test irrelevant thinking (β = 0.403 p < 0.001) is positive and significantly related to teacher avoidance for examination anxiety.

**Table 5:** Summary of the hypotheses testing.

Effect	Original coefficient	t-value	p-value (2-sided)

GTW -> TOP	0.126	2.917	0.004
GTW -> AVO	–0.052	–1.034	0.301
GTW -> SSS	0.145	2.699	0.007
EMO -> TOP	0.452	8.773	0.000
EMO -> AVO	0.274	5.150	0.000
EMO -> SSS	0.205	3.427	0.001
TIT -> TOP	–0.195	–3.604	0.000
TIT -> AVO	0.403	6.841	0.000
TIT -> SSS	0.042	0.760	0.447

**Figure 1:**
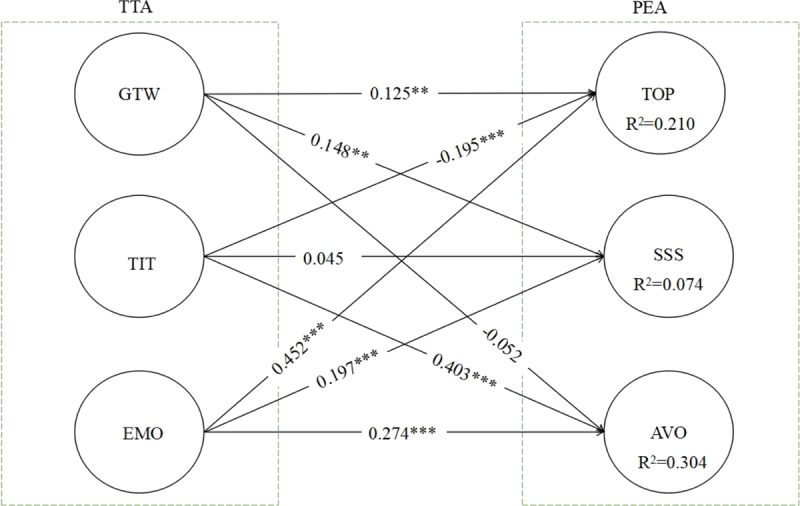
Structural model testing.

Moreover, the study perceived a negatively significant effect between general test worrying (β = –0.052) and teacher avoidance of pre-examination anxiety. General test worrying is positive and significantly related to teachers seeking social support for taking pre-exam anxiety (β = 0.145, p < 0.001); emotion (β = 0.205, p < 0.001) is positively and significantly associated with teacher’s seeking social support for taking pre-examination anxiety. The result reveals an insignificant relationship between the test irrelevant thinking (β = 0.042, p = 0.447) and teachers seeking social support for pre-examination anxiety. The structural model testing results indicate in Figure 1. As R-Square (R^2^) indicates that the predictor constructs (general test worrying, emotion, and test irrelevant thinking) explained 21% of teacher task-orientation and preparation of pre-exam anxiety and 30% for the avoidance of pre-exam anxiety respectively.

## Discussion and Implications

The study tested linear relations and model fits to confirm teacher’s perspectives on examination anxiety and strategic approach towards pre-exam anxiety as well as the relationships between specific causes of examination anxiety and dimensions of coping with pre-exam anxiety among teachers which is in line with previous studies (Senler, 2016; Von der Embse et al., 2017). Previous studies acknowledged a significant relationship between causes and dimensions of coping with pre-exam anxiety among teachers (Von der Embse et al., 2017; Contreras-Soto et al., 2019). Therefore, this study findings further add to the body of knowledge by providing a more detailed explanation and propose a model for causes of teacher examination anxiety and dimensions of coping with pre-exam anxiety to which factors of anxiety are interconnected with one another as indicated by six key factors of teacher exam anxiety and the dimensions of coping with pre-exam anxiety in the current study.

Furthermore, the relationship between causes of examination anxiety and pre-exam anxiety among teachers as the results indicate an increase in general test worrying, the more task – orientation and more preparation for exam similar to Arana and Furlan (2016) findings that peoples’ personal worry helps in maintaining task – orientation and preparation effectively with supplementary psychological features for efficiency during the examination.

In addition, the positive and significant effect of general test worrying helps teacher’s seeking social support from people around. Based on these current examination worry issues, educators are indeed helped to gain more and positive experiences (Ho, 2015). Therefore, factors of examination anxiety such as worry, tension, and bodily symptoms components are significant to social support, and avoidance.

The results also show that positive emotions increase task – orientation and preparation for examinations. Specifically, positive emotions such as happiness, confidence, euphoria, and interest predicted task – orientation and preparation for examinations are critical to educational development (Doron, Stephan, Maiano & Le Scanff, 2011; Ketonen, Malmberg, Salmela-Aro, Muukkonen, Tuominen & Lonka, 2018).

Also, emotions predicts teacher’s seeking social support; as it proves to be relevant factor in pre-test anxiety especially on avoidance. According to Thomas and Gadbois (2007) self-handicapping (procrastination, avoidance and strategic withdrawal of effort) is positively related to test anxiety. Therefore, these results align with relationships between causes of test anxiety and dimensions of coping with pre-examination anxiety factors.

However, teacher’s irrelevant thinking supports correlative relationship with task – orientation and preparation; as part of cognitive anxiety such as task – orientation and preparation; and teacher’s irrelevant thinking are among components of anxiety that are consistent with the theoretical framework of anxiety (Danthony, Mascret & Cury, 2019).

Also, the present study identifies negative findings: the relationship between teacher’s irrelevant thinking and teachers seeking social support, which is contrary to the practice aforesaid. The result confirmed a negative impact of teacher irrelevant thinking towards seeking social support from others and test-irrelevant thoughts indicate insignificant effect on seeking social support from others. The result also identified a strong relationship between general test worry and other factors of test anxiety that have been found to provide a positive correlation with avoidance coping strategy (Putwain et al., 2012). Nonetheless, the results are inconsistent towards measuring general coping strategies, especially focusing on coping with examination pressure and measuring other forms of coping strategies when testing for pre, during and post stages of anxiety (Putwain et al., 2012; Putwain et al., 2016).

Extensively, these findings provide theoretical evidence on teacher exam anxiety as one of the subject matter that helps improve teachers’ skill and competence development especially in educational environments. The knowledge of teacher test anxiety is susceptible to social activities and established different activities within educational environment (Von der Embse et al., 2015), but the causes and dimensions of coping with pre-exam anxiety among teachers might experience inconsistent anticipated results. For example in the local level, teachers lack proper awareness can be the causes of anxiety and then it turns to problem-solving opportunities to lower the level of test anxiety in the global scale. In the global scale, educational administrators and policy-makers should improve on diverse methods of anxiety to help in reconstructing and combating test anxiety in educational settings particularly educational and professional development in the teacher and teaching profession.

## Limitations and future directions

One of the study limitations is the use of self-report data which might produce a bias such as the ability for respondents unable to answer accurately, available populations unwilling to participate in the survey and broad instrument of survey research might reflect on the outcomes of the results (MacKenzie & Podsakoff, 2012). Furthermore, further studies may focus on qualitative analysis – through teacher detail experiences or phenomenological approach to capture the core objective of examination anxiety among teachers.

Second, the use of a cross-sectional approach for this study cannot be generalized based on constructs used for the study, that is the mutual relationship that exists between examination anxiety and predicting the dimensions of coping with pre-examination anxiety among teachers. Therefore, other approaches such as longitudinal studies are needed to explore unique dynamics and trends on relationships between cause and pre-anxiety among teachers in future work.

Also, psychological improvement in the theoretical knowledge on the cause and dimensions of coping with pre-exam anxiety among teachers might provide a better understanding of teachers’ anxiety and the development of teacher anxiety interventions through contributions towards research on issues concerning the diverse forms of anxiety among teachers as added knowledge to the theory of psychology. Further, teachers’ preparation for examinations, coupled with dimensions and dimensions of coping with anxiety remains complex tasks that need multiple knowledge from counsellor, psychology and educational consortium to enormously involve in finding solutions to anxiety among teachers.

Finally, the undivided attention of counselling psychologists should be subtly engaged to teacher examination anxiety and the specific need to support practical strategies that positively enhance program adoption and advocate for the value, to increase the level of teaching competencies, and to standardize the established teaching profession.
